# The effect of propranolol on the prognosis of hepatocellular carcinoma: A nationwide population-based study

**DOI:** 10.1371/journal.pone.0216828

**Published:** 2019-05-24

**Authors:** Ping-Ying Chang, Chi-Hsiang Chung, Wei-Chou Chang, Chun-Shu Lin, Hsuan-Hwai Lin, Ming-Shen Dai, Ching-Liang Ho, Wu-Chien Chien

**Affiliations:** 1 Division of Hematology/Oncology, Department of Internal Medicine, Tri-Service General Hospital, National Defense Medical Center, Taipei, Taiwan, Republic of China; 2 School of Public Health, National Defense Medical Center, Taipei, Taiwan, Republic of China; 3 Department of Medical Research, Tri-Service General Hospital, National Defense Medical Center, Taipei, Taiwan, Republic of China; 4 Department of Radiology, Tri-Service General Hospital, National Defense Medical Center, Taipei, Taiwan, Republic of China; 5 Department of Radiation Oncology, Tri-Service General Hospital, National Defense Medical Center, Taipei, Taiwan, Republic of China; 6 Division of Gastroenterology, Department of Internal Medicine, Tri-Service General Hospital, National Defense Medical Center, Taipei, Taiwan, Republic of China; Chang Gung Memorial Hospital at Linkou, TAIWAN

## Abstract

**Background:**

Beta-blockers can reduce recurrence, metastasis, and mortality in various cancers. In this study, we investigated the effect of propranolol, a non-selective beta-blocker on overall survival (OS) in unresectable/metastatic hepatocellular carcinoma (HCC) and on recurrence-free survival (RFS) in resectable, curable HCC.

**Methods:**

Data were retrieved from the Taiwan National Health Insurance Research Database between January 2000 and December 2013. Propranolol users (for >1 year) and non-propranolol users were matched using a 1:2 propensity score in both cohorts.

**Results:**

The unresectable/metastatic HCC cohort comprised 1,560 propranolol users and 3,120 non-propranolol users (control group). On multivariate Cox regression analysis of HCC mortality, propranolol significantly reduced the mortality risk by 22% (hazard ratio [HR] = 0.78, 95% confidence interval [CI] 0.72–0.84, P <0.001). On stratified Cox regression analysis, propranolol also reduced the mortality risk in HCC patients with hepatitis B (HR = 0.92, 95% CI 0.85–0.99, P = 0.045), hepatitis C (HR = 0.85, 95% CI = 0.78–0.92, P = 0.001), liver cirrhosis (HR = 0.78, 95% CI = 0.72–0.85, P <0.001), and diabetes mellitus (HR = 0.87, 95% CI = 0.81–0.94, P = 0.008). The resectable, curable HCC cohort comprised 289 propranolol users and 578 non-propranolol users (control group), but there was no significant difference in RFS (P = 0.762) between propranolol and non-propranolol users.

**Conclusion:**

This study revealed that propranolol could improve OS in unresectable/metastatic HCC.

## Introduction

Hepatocellular carcinoma (HCC) is the sixth most common cancer and the third leading cause of cancer mortality worldwide.[1] Approximately 80% of HCC cases occur in eastern Asia and the sub-Saharan Africa, with chronic hepatitis B virus (HBV) infection being the most predominant risk factor.[2] In Western countries, hepatitis C virus (HCV) infection and excessive alcohol consumption are important risk factors.[2, 3] The treatment strategies for HCC include surgery (liver resection [LR] and liver transplantation [LT]), locoregional treatment (radiofrequency ablation [RFA] and transarterial chemoembolization [TACE]), and use of multikinase inhibitors (sorafenib and regorafenib).[4] LR has been the most effective treatment for HCC, and the 5-year survival is 38–61% with different stages.[5] However, <30% of HCC patients are eligible for surgery.[6] Furthermore, recurrence after LR occurs in up to 80% of patients at 5 years.[5, 7] Meanwhile, TACE is used for patients with preserved liver function who are ineligible for LR (with asymptomatic multifocal or bulky tumor).[8] For advanced-stage HCC, sorafenib has been approved as the standard first-line treatment and regorafenib as second-line treatment.[9, 10] However, the treatment outcomes remain unsatisfactory.

The beta adrenergic receptor (ADRB) pathway is essential for normal physiologic functions. Beta-2 adrenergic receptor (ADRB2) signaling regulates multiple signaling cascades involved in cancer progression and metastasis, including proliferation and invasion of cancer cells and angiogenesis, through activation of the cyclic adenosine monophosphate/protein kinase A pathway.[11–13] Propranolol, a nonselective beta-blocker (NSBB), has shown anticancer effects in preclinical studies.[14, 15] Beta-blockers have been demonstrated to reduce metastasis, recurrence, and mortality in various types of cancer.[16–19] Several retrospective studies have shown that beta-blockers can reduce cancer risk.[20, 21] Furthermore, ADRB2 expression has been reported to be upregulated in HCC,[22] which may help explain the capability of propranolol to reduce HCC risk in patients with cirrhosis.[23, 24]

Using a large data set available in Taiwan, we conducted this nationwide population-based cohort study to clarify the effect of propranolol on the prognosis of unresectable/metastatic and resectable, curable HCC.

## Materials and methods

### Data source

Data were extracted from the National Health Insurance Research Database (NHIRD) of Taiwan. The Taiwan National Health Insurance (NHI) program covers >99% of the 23 million residents of Taiwan. The data in this study were specifically obtained from the Longitudinal Health Insurance Database (LHID), a subset of the NHIRD. The LHID, which consists of comprehensive information such as demographic data, dates of clinical visits, and disease diagnoses for 1 million enrollees, is derived from the medical claims records of the NHI program. The diagnostic codes are based on the International Classification of Diseases, Ninth Revision, Clinical Modification (ICD-9-CM). The Registry for Catastrophic Illness Patient Database (RCIPD) is also a subset of the NHIRD and includes data from insured residents with severe diseases defined by the NHI program, such as malignancies. The patients included in the RCIPD comprised the case group in this study. The Institutional Review Board of the Tri-Service General Hospital (TSGHIRB no. 2-105-05-082) approved the study and waived the requirement of written informed consent All methods were performed in accordance with the relevant guidelines and regulations.

### Study population and definition of propranolol exposure

Between January 1, 2000, and December 31, 2013, we extracted data from the LHID and RCIPD for patients aged ≥20 years with complete age and sex information and with a history of HCC (ICD-9-CM codes 155.0 and 155.2). Patients with a diagnosis of malignancies (ICD-9-CM codes 140–209) other than HCC before study enrollment were excluded.

To investigate the role of propranolol in unresectable/metastatic HCC, we selected patients who used propranolol, and the index date was the initial date of palliative treatment with TACE, radiotherapy, chemotherapy, or sorafenib. Those who expired before enrollment were excluded. We also studied the effect of propranolol on resectable, curable HCC, and the index date was the initial date of curative hepatic surgery. Patients who had undergone a hepatic operation such as lobectomy (ICD-9-CM code 50.3), segmentectomy (ICD-9-CM code 50.22), hepatectomy (partial, ICD-9-CM code 50.4; total, ICD-9-CM code 50.22), or LT (ICD-9-CM code 50.59) before enrollment were also excluded. Moreover, patients were excluded if they received any treatment before the curative operation, including TACE (ICD-9-CM codes 99.25 + 88.47), RFA (ICD-9-CM codes 50.29 + 88.76), radiotherapy (ICD-9-CM codes 92.3x), chemotherapy (including doxorubicin, fluorouracil, gemcitabine, oxaliplatin, or cisplatin), and sorafenib.

In both cohorts, patients were divided into two groups according to propranolol exposure: a propranolol group, consisting of patients who underwent propranolol therapy for at least 1 year before enrollment and had no prescription for another beta-blocker during that time, and a non-propranolol group, consisting of patients who did not receive propranolol therapy.

We used the defined daily dose (DDD), which is recommended by the World Health Organization (http://www.whocc.no/atc_ddd_index/), to measure the prescribed drug amount. DDD is the assumed average maintenance daily dose of a drug consumed for its main indication in adults. The recommended DDD of propranolol in this study was based on the treatment of mild-moderate hypertension. To examine the dose-response relationship, we categorized the propranolol cohorts into three groups (<545, 545–730, and >730 cumulative DDD).

### Propensity score matching and comorbidities

To reduce selection bias, the propranolol and non-propranolol groups were selected according to twofold propensity score matching in studies of advanced/metastatic and surgically curable HCC. The propensity score was calculated using logistic regression to estimate the probability of treatment assignment according to baseline variables, including age, sex, Charlson comorbidity index score, diabetes mellitus (DM; ICD-9-CM code 250), HBV (ICD-9-CM codes V02.61, 070.20, 070.22, 070.30, and 070.32), HCV (ICD-9-CM codes V02.62, 070.41, 070.44, 070.51, 070.54, and 070.7), alcoholic liver disease (ICD-9-CM codes A213, A215, A347, 291, 303, 305, 571.0, 571.1, 571.2, and 571.3), liver cirrhosis (ICD-9-CM codes 571.2, 571.5, 571.6, 572.2, 572.3, 572.4, 572.8, and 573.0), renal failure (ICD-9-CM codes 584–586), hypertension (ICD-9-CM codes 401–405), coronary artery disease (ICD-9-CM codes 410–414), and medications of metformin, statins, aspirin, thiazolidinediones (TZDs), fibrates, and angiotensin-converting enzyme inhibitors (ACEIs).

### Study outcomes

In the unresectable/metastatic HCC cohort, the study outcome was overall survival (OS), whereas in the resectable, curable HCC cohort, the study outcome was recurrence-free survival (RFS). HCC recurrence was defined as a repeat of any treatment including LR, LT, TACE, or RFA for HCC during the observation period. All subjects were followed from the index date until the occurrence of events or the end of 2013.

### Statistical analysis

Continuous data are shown as the mean ± standard deviation (SD) and categorical data are shown as the frequency and percentage (%) of each baseline characteristic. Chi-squared test is used to evaluate the distributions of demographic features and common comorbidities between propranolol and non-propranolol groups. The mean ages of the cohorts were compared using a Mann-Whitney U-test. The Kaplan-Meier method was employed to plot the cumulative curves of disease recurrence and mortality, and the log-rank test was performed to examine the difference between the curves. A Cox regression model with stratification was used to evaluate the relationships between propranolol use and potential risk factors in HCC patients. Furthermore, hazard ratios (HRs) and 95% confidence intervals (CIs) for the outcomes were calculated between propranolol and non-propranolol users after adjusting for variables. SAS version 9.4 (SAS Institute, Cary, NC, USA) was used for all statistical analyses. A P value of <0.05 was considered statistically significant.

## Results

### Propranolol therapy was associated with better OS in unresectable/metastatic HCC

**Fig 1:**
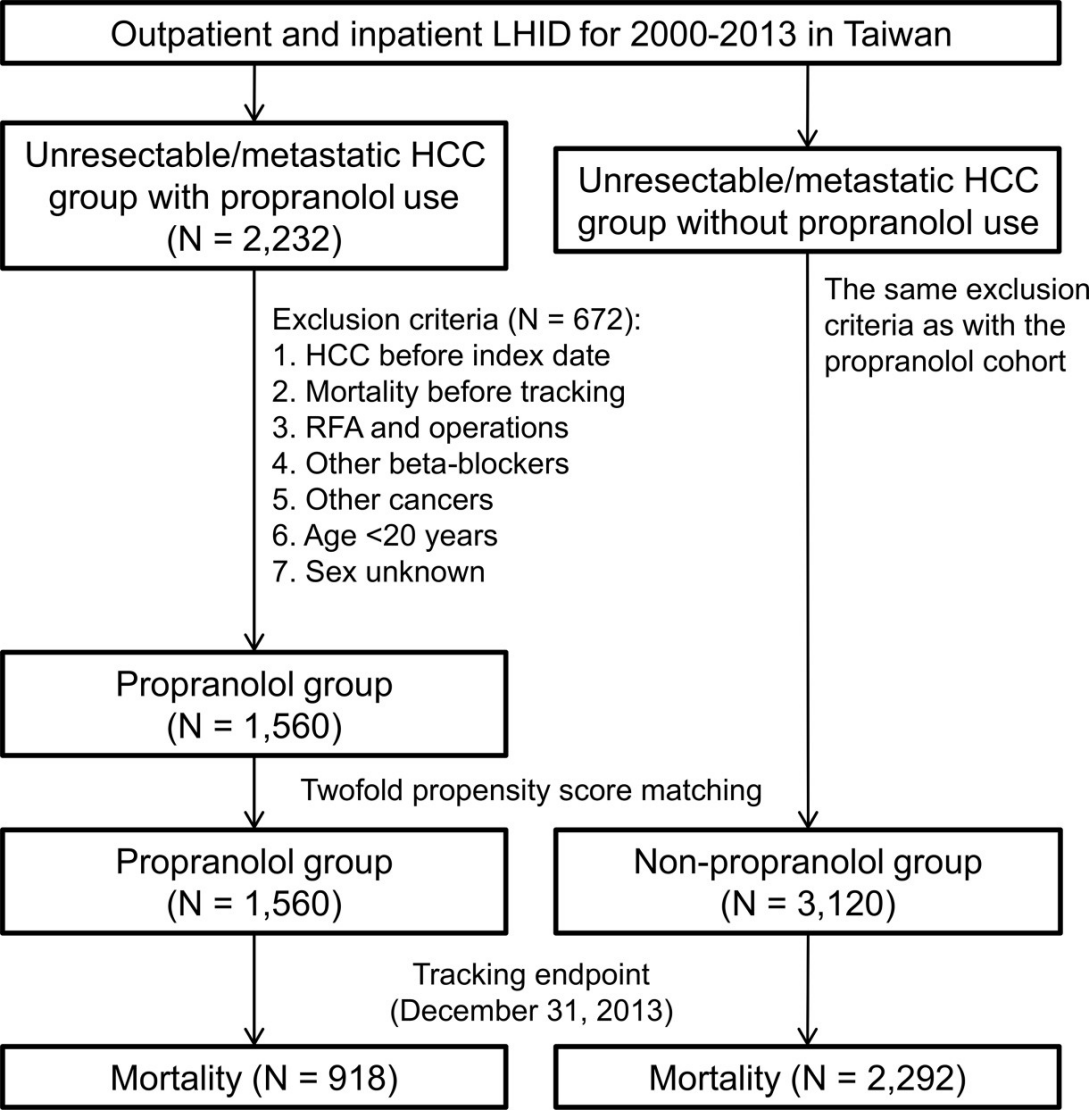
Flowchart for matching patients with unresectable/metastatic HCC according to propranolol exposure. LHID, Longitudinal Health Insurance Database; HCC, hepatocellular carcinoma; RFA, radiofrequency ablation. Operations include lobectomy, segmentectomy, hepatectomy, and liver transplantation.

We identified 2,232 patients with unresectable/metastatic HCC who used propranolol for at least 1 year before enrollment. Among them, 672 patients were excluded after the inclusion and exclusion criteria were applied. We also randomly selected 3,120 HCC patients (matched with twofold propensity score matching) who did not use propranolol as control (Fig 1). The baseline clinical characteristics between the propranolol and non-propranolol groups showed no significant difference (Table 1).

**Table 1:** Demographic characteristics and comorbidities of patients with unresectable/metastatic HCC according to propranolol use after propensity score matching.

Variable	Propranolol		Non-propranolol		P
	n	%	n	%	
**Total**	1,560	33.3	3,120	66.7	
**Sex**					
** Male**	1,151	73.8	2,302	73.8	1.00
** Female**	409	26.2	818	26.2	
**Age, years, (mean ±SD)**	63.0 ± 12.7		62.5 ± 12.6		0.65
** 20–49**	250	16.0	500	16.0	1.00
** 50–64**	559	35.8	1,118	35.8	
** ≥65**	751	48.1	1,502	48.1	
**Comorbidity**					
** HBV**	440	28.3	910	29.3	0.49
** HCV**	352	22.6	720	23.1	0.71
** Alcoholic liver disease**	93	6.0	201	6.4	0.57
** Liver cirrhosis**	954	61.2	1,923	61.6	0.75
** Renal failure**	65	4.2	129	4.1	0.96
** DM**	278	17.8	525	16.8	0.41
** HTN**	249	16.0	459	14.7	0.26
** CAD**	39	2.5	71	2.3	0.68
** CCI_R, (mean ±SD)**	0.5 ± 1.1		0.6 ± 1.2		0.12
**Medication**					
** Aspirin**	292	18.7	581	18.6	0.94
** Statins**	19	1.2	40	1.3	0.89
** Metformin**	142	9.1	282	9.0	0.96
** Lipid-lowering drugs**	190	12.2	377	12.1	0.92
** TZDs**	186	11.9	365	11.7	0.85
** ACEIs**	50	3.2	103	3.3	0.93
**Treatment**					
** Sorafenib**	711	45.6	1,419	45.5	0.95
** TACE**	765	49.0	1,533	49.1	0.95
** Radiotherapy**	1,249	80.1	1,556	81.9	0.12

HCC, hepatocellular carcinoma; HBV, hepatitis B virus; HCV, hepatitis C virus; DM, diabetes mellitus; HTN, hypertension; CAD, coronary artery disease; CCI_R, Charlson comorbidity index after removal of the above mentioned comorbidities; TZDs, thiazolidinediones; ACEIs, angiotensin-converting enzyme inhibitors; TACE, transarterial chemoembolization

**Fig 2:**
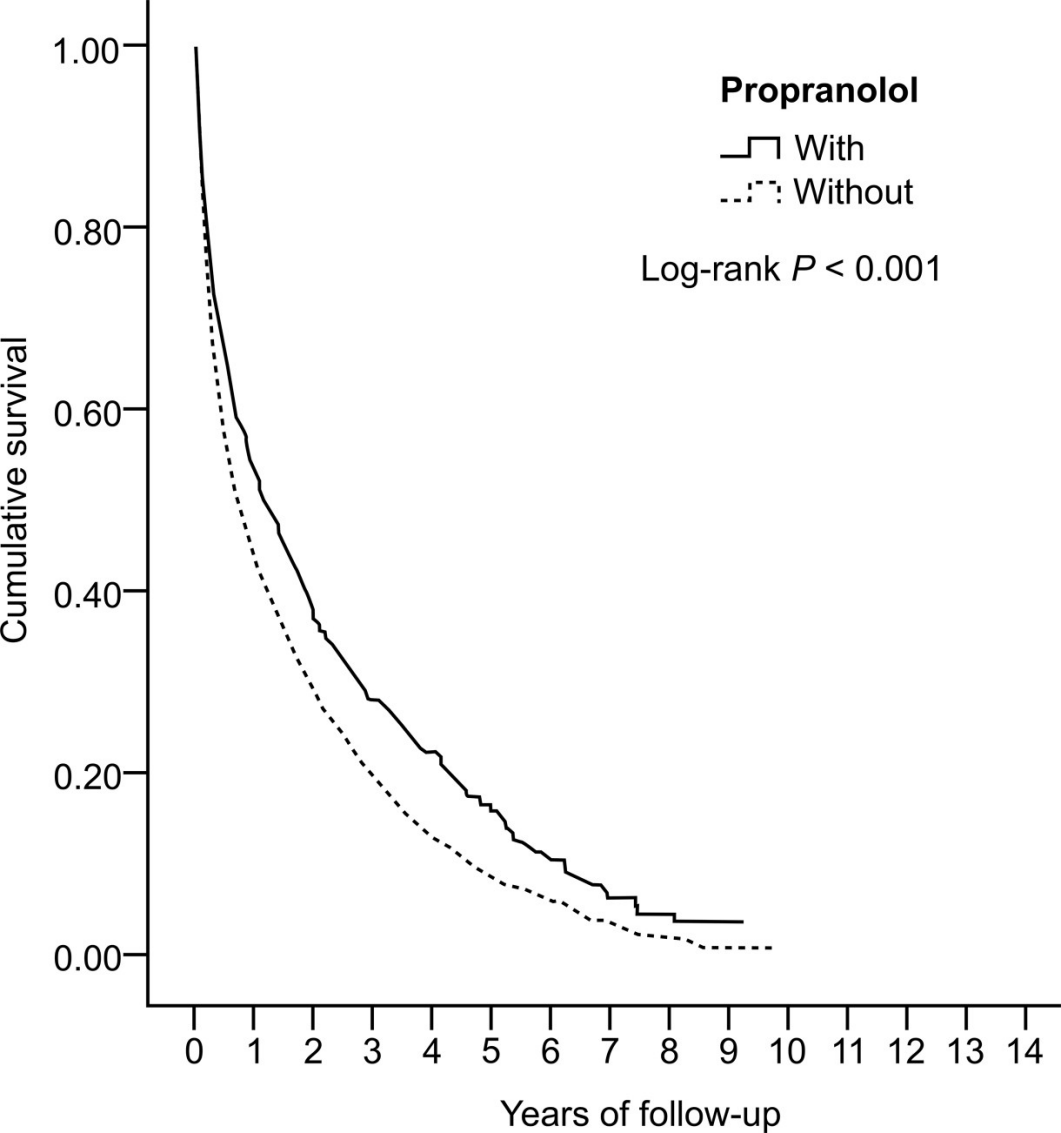
Kaplan-Meier plot for the probability of overall survival in patients with unresectable/metastatic hepatocellular carcinoma according to propranolol exposure.

In the Kaplan-Meier analysis for OS, the propranolol group had higher survival than the non-propranolol group at the end of follow-up (41.2% vs. 26.5%, P < 0.001, Fig 2). In the Cox regression multivariate analysis of HCC mortality, propranolol significantly reduced the mortality risk by 22% (HR = 0.78, 95% CI = 0.72–0.84, P < 0.001). Males had a higher risk of mortality than females (HR = 1.14, 95% CI = 1.05–1.24, P < 0.001). Mortality risk decreased significantly with longer duration of propranolol use. Moreover, patients with increasing age showed a higher risk of mortality. HBV and HCV infection also increased the mortality risk (HR = 1.08 [95% CI = 1.01–1.18, P = 0.04) and 1.36 [95% CI = 1.17–1.50], respectively, P < 0.001). Meanwhile, the use of aspirin, statins, metformin, fibrates, TZDs, and ACEIs did not influence the mortality risk. These findings are shown in Table 2.

**Table 2:** Univariate and multivariate Cox regression analyses of mortality in patients with unresectable/metastatic HCC.

Variable	Crude HR (95% CI)	P	Adjusted HR (95% CI)	P
**Propranolol**				
** **With	0.77 (0.72–0.83)	<0.001*	0.78 (0.72–0.84)	<0.001*
** **Without	Reference 1.00		Reference 1.00	
**Propranolol**				
** **<545 days	0.81 (0.73–0.99)	0.04*	0.81 (0.74–0.99)	0.045*
** **545–730 days	0.77 (0.70–0.83)	0.001*	0.78 (0.71–0.84)	0.002*
** **>730 days	0.69 (0.58–0.79)	<0.001*	0.70 (0.59–0.80)	<0.001*
** **Without	Reference 1.00		Reference 1.00	
**Sex**				
** **Male	1.20 (1.10–1.29)	<0.001*	1.14 (1.05–1.24)	<0.001*
** **Female	Reference 1.00		Reference 1.00	
**Age group (years)**				
** **≥65	1.41 (1.27–1.56)	<0.001*	1.13 (1.06–1.88)	<0.001*
** **50–64	1.24 (1.15–1.34)	<0.001*	1.09 (1.01–1.29)	0.03*
** **20–49	Reference 1.00		Reference 1.00	
**HBV infection**				
** **With	1.09 (1.01–1.20)	0.04*	1.08 (1.01–1.18)	0.04*
** **Without	Reference 1.00		Reference 1.00	
**HCV infection**				
** **With	1.45 (1.29–1.63)	<0.001*	1.36 (1.17–1.50)	<0.001*
** **Without	Reference 1.00		Reference 1.00	
**Alcoholic liver disease**				
** **With	1.04 (0.91–1.20)	0.56	1.08 (0.76–1.12)	0.10
** **Without	Reference 1.00		Reference 1.00	
**Liver cirrhosis**				
** **With	1.07 (1.01–1.15)	0.045*	1.06 (0.99–1.15)	0.12
** **Without	Reference 1.00		Reference 1.00	
**Renal failure**				
** **With	1.12 (0.94–1.33)	0.21	1.11 (0.94–1.32)	0.23
** **Without	Reference 1.00		Reference 1.00	
**DM**				
** **With	1.01 (0.83–1.00)	0.05	1.07 (0.89–1.08)	0.46
** **Without	Reference 1.00		Reference 1.00	
**HTN**				
** **With	1.81 (1.74–1.90)	<0.001*	1.01 (0.82–1.01)	0.08
** **Without	Reference 1.00		Reference 1.00	
**CAD**				
** **With	1.00 (0.79–1.25)	0.95	1.07 (0.86–1.37)	0.49
** **Without	Reference 1.00		Reference 1.00	
**CCI_R**	1.05 (1.02–1.09)	0.001*	1.03 (0.99–1.06)	0.12
**Aspirin**				
** **With	1.27 (0.64–1.91)	0.45	1.25 (0.66–1.97)	0.45
** **Without	Reference 1.00		Reference 1.00	
**Statins**				
** **With	1.14 (0.88–1.80)	0.29	1.11 (0.83–1.86)	0.30
** **Without	Reference 1.00		Reference 1.00	
**Metformin**				
** **With	1.10 (0.75–1.39)	0.34	1.10 (0.77–1.34)	0.34
** **Without	Reference 1.00		Reference 1.00	
**Fibrates**				
** **With	1.45 (0.67–2.19)	0.39	1.35 (0.67–2.03)	0.40
** **Without	Reference 1.00		Reference 1.00	
**TZDs**				
** **With	1.20 (0.52–1.60)	0.48	1.12 (0.50–1.60)	0.48
** **Without	Reference 1.00		Reference 1.00	
**ACEIs**				
** **With	1.10 (0.83–1.56)	0.67	1.08 (0.85–1.46)	0.60
** **Without	Reference 1.00		Reference 1.00	

*Significantly correlated with outcome, P-value < 0.05. HCC, hepatocellular carcinoma; HR, hazard ratio; CI, confidence interval; HBV, hepatitis B virus; HCV, hepatitis C virus; DM, diabetes mellitus; HTN, hypertension; CAD, coronary artery disease; CCI_R, Charlson comorbidity index after removal of the above mentioned comorbidities; TZDs, thiazolidinediones; ACEIs, angiotensin-converting enzyme inhibitors

We used stratified Cox regression analysis to evaluate the effect of propranolol on each parameter. Propranolol reduced the mortality risk in both males and females and significantly in all age groups. Propranolol also reduced the mortality risk in HCC patients with HBV infection (HR = 0.92, 95% CI = 0.85–0.99, P = 0.045), HCV infection (HR = 0.85, 95% CI = 0.78–0.92, P = 0.001), liver cirrhosis (HR = 0.78, 95% CI = 0.72–0.85, P < 0.001), and DM (HR = 0.87, 95% CI = 0.81–0.94, P = 0.008). Propranolol significantly lowered the mortality risk in patients who used and did not use aspirin, metformin, fibrates, and ACEIs (S1 Table).

We further analyzed the effect of palliative treatment (sorafenib, TACE, or radiotherapy) with and without propranolol use on HCC mortality. In patients with palliative treatment, those who received propranolol had a lower risk of mortality than those who did not receive propranolol (S2 Table).

### Propranolol therapy did not improve RFS in resectable, curable HCC

**Fig 3:**
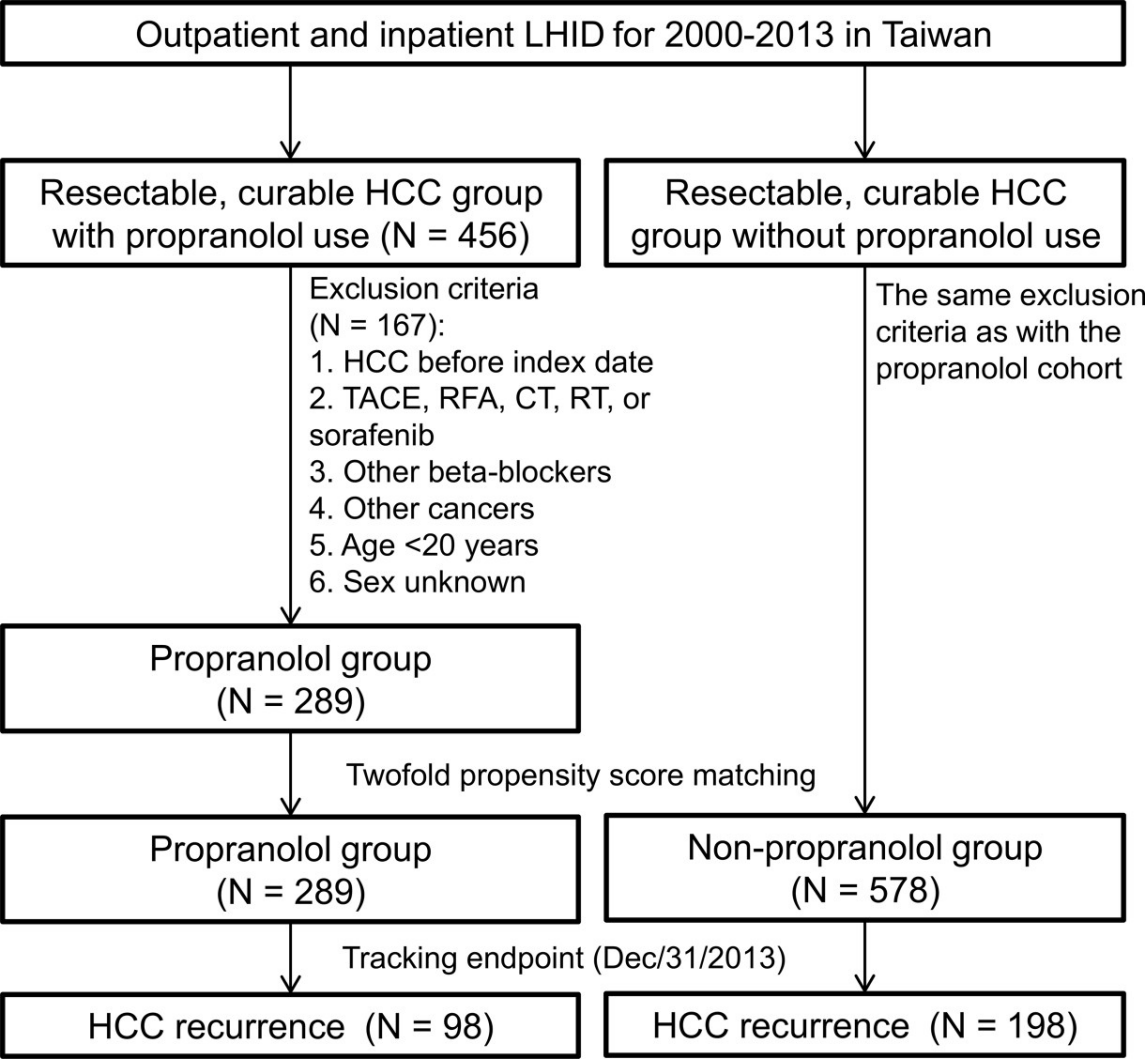
Flowchart for matching patients with resectable, curable HCC according to propranolol exposure. LHID, Longitudinal Health Insurance Database; HCC, hepatocellular carcinoma; TACE, transarterial chemoembolization; RFA, radiofrequency ablation; CT, chemotherapy; RT, radiotherapy.

**Fig 4:**
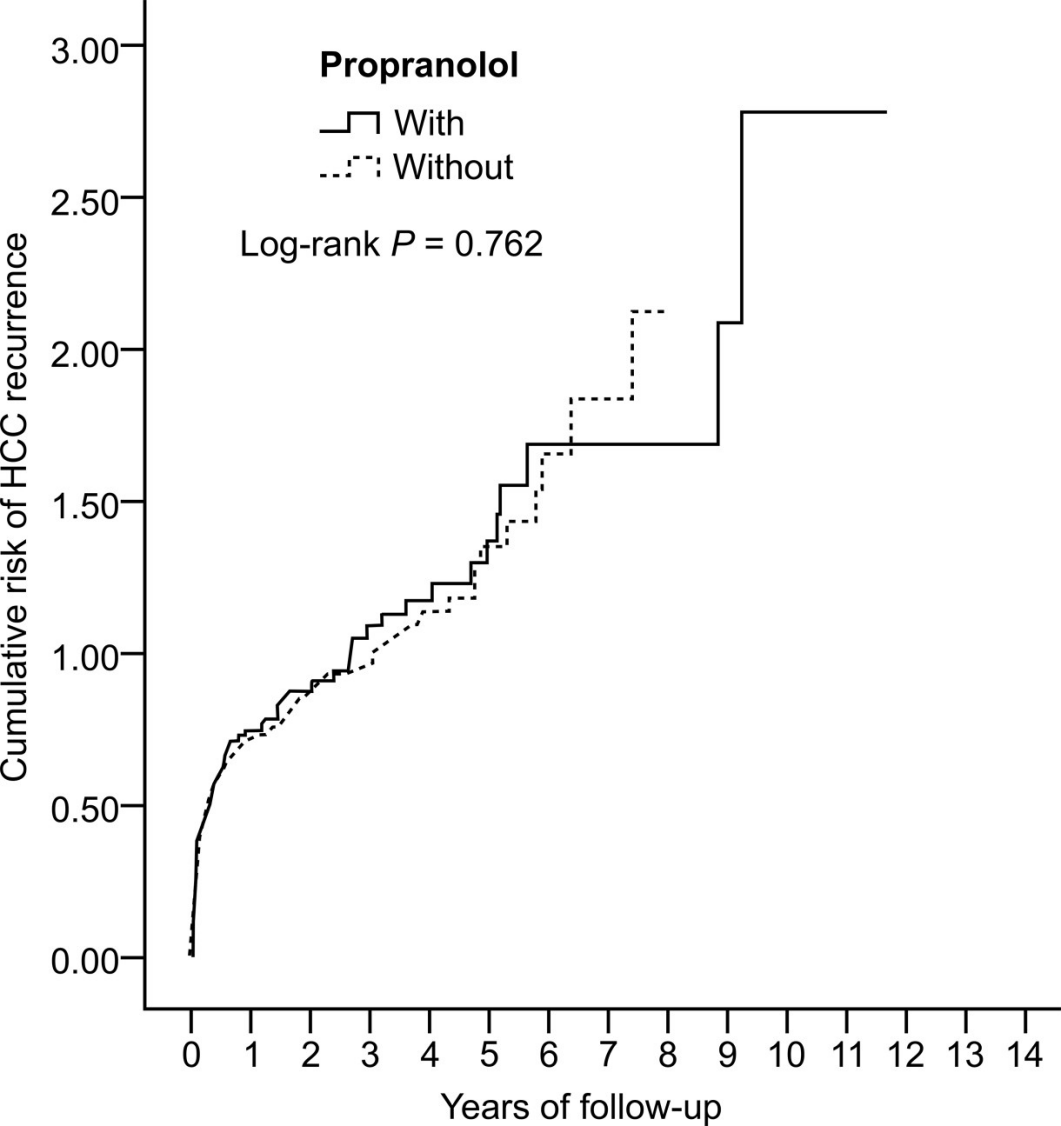
Kaplan-Meier plot for the probability of recurrence of resectable, curable hepatocellular carcinoma according to propranolol exposure.

We identified 456 patients with resectable, curable HCC who received propranolol for at least 1 year before enrollment. Among them, 167 patients were excluded after the inclusion and exclusion criteria were applied. We also randomly selected 578 HCC patients (matched with twofold propensity score matching) who did not receive propranolol as control (Fig 3). RFS was not significantly different between propranolol and non-propranolol users (98/289 = 33.9% vs. 198/578 = 34.3%, P = 0.762, Fig 4). Events of early recurrence (≤2 years) (82/289 = 28.4% vs. 179/578 = 31.0%, P = 0.166) and late recurrence (>2 years) (16/289 = 5.5% vs. 19/578 = 3.3%, P = 0.780) were also not significantly different between propranolol and non-propranolol users.

## Discussion

Our study showed that propranolol can reduce the mortality risk by 22% in unresectable/metastatic HCC. The risk was reduced in both sexes and all age groups. Propranolol also significantly reduced the mortality risk in patients with HBV and HCV infection, liver cirrhosis, and DM. There was also risk reduction with longer duration of propranolol use. Additionally, propranolol treatment could reduce the mortality risk in HCC patients regardless of aspirin, metformin, fibrate, and ACEI use. However, propranolol did not improve RFS in resectable, curable HCC.

Extensive preclinical data have firmly established the relevance of the ADRB signaling system in cancer biology, whose effects are mediated mainly through ADRB2 activation of the protein kinase A pathway.[11–13] Several retrospective studies have examined the influence of beta-blockers on cancer survival.[16–19, 25–29] However, some studies support the protective effect of beta-blockers,[16–19] while others do not.[25–29] In most of the studies, beta-blockers were not categorized according to their beta-1 or beta-2 selectivity. The similarity between ADRB1 and ADRB2 and the affinity of beta-blockers toward these receptors could explain the confusing results in such studies.[30] Furthermore, NSBBs have been shown to have more survival benefit than selective beta-blockers in ovarian cancer.[18]

Liver cirrhosis is the long-term complication of chronic hepatitis, and most HCCs develop in cirrhotic livers. Serum levels of catecholamines in patients with cirrhotic livers have been reported to be associated with severity of liver disease.[31] NSBBs are recommended in patients with liver cirrhosis and esophageal varices for bleeding prophylaxis. Long-term use of propranolol, an NSBB, has been demonstrated to reduce the risk of HCC developing in patients with HCV-associated cirrhosis.[23] A meta-analysis also showed that NSBBs may reduce HCC risk in patients with cirrhosis.[24] These findings are consistent with the findings in our study, which revealed that propranolol could reduce the mortality risk in patients with HBV and HCV infection, liver cirrhosis, and DM, and the protective effect was more substantial when the propranolol exposure was longer. Aspirin, statins, TZDs, and metformin are known for their anticancer effects,[32] while ACEIs and fibrates have equivocal effects on cancer risk and survival.[33, 34] However, in our study, propranolol was an independent prognostic factor for mortality in the Cox regression analysis, while the above candidate medications were not. Furthermore, in the stratified Cox regression analysis, propranolol could lower the mortality risk in patients who did not use these candidate medications.

In the joint analysis, we found that propranolol may have a synergistic effect with sorafenib, radiotherapy, and TACE. Sorafenib is currently the standard treatment for metastatic HCC, but the development of acquired resistance is almost inevitable with this drug. The phosphatidylinositol-3-kinase/protein kinase B pathway, autophagy, epithelial-mesenchymal transition, epigenetic regulation, and tumor environment are involved in the resistance mechanism.[35] Interestingly, ADRB2 signaling blockade by propranolol has been demonstrated to enhance the autophagic degradation of hypoxia-inducible factor 1-alpha, thereby enhancing sorafenib efficacy.[36] Moreover, beta-blocker use during definitive radiotherapy has been reported to improve survival in patients with non-small cell lung cancer.[37] ADRB2 signaling promotes angiogenesis through crosstalk between endothelial and cancer cells.[38] ADRB2s also play a critical role in endothelial cell proliferation, revascularization, and neoangiogenesis in response to ischemia.[39] Therefore, the combination of NSBBs and TACE may achieve a more effective antitumor effect.

LR and LT are some of the treatment options for HCC. However, recurrence is not uncommon after LR and is the leading cause of postoperative death.[5, 6] The early recurrence of HCC has been reported to be within 2 years after LR and is related to micrometastasis, whereas the late recurrence has been reported to occur >2 years after LR, which is attributed to the de novo tumor.[7] Clinicopathologic parameters such as tumor stage, vascular invasion, alpha-fetoprotein, liver cirrhosis, multi-nodularity, and grade of hepatitis activities are known predictors of recurrence.[7, 40, 41] Several adjuvant treatments including interferon, chemotherapy, and sorafenib have been investigated in clinical trials, but none of the studies reported successful outcomes except with interferon therapy, which prolonged the survival in specific patient groups.[42] In our study, propranolol did not improve RFS in resectable, curable HCC. There was also no significant difference in the risk of early or late recurrence between propranolol and non-propranolol users. Because the NHIRD did not provide clinicopathologic data, we could not use these parameters for further analysis. ADRB2 expression in HCC tissues is associated with several poor prognostic factors and is an independent prognostic factor for RFS and OS in surgically curable HCC.[43, 44] Hence, immunohistochemical staining of ADRB2 expression may provide useful information for considering NSBB treatment.

This study has several limitations. First, the clinicopathologic and laboratory parameters of enrolled patients were not available from the NHIRD, and we could not classify the patients according to individual prognostic factors. Propensity score matching and multivariate analysis were used to adjust for potential confounders. Second, we could not measure the actual dose of propranolol and duration of use. We presumed that all medications were taken by the patients as prescribed. The DDD was calculated based on official prescriptions and we could not investigate the dose-response effect. Finally, the incidence of HCC recurrence might have been underestimated. However, this problem would have occurred in both matched groups and might have a limited impact on our results.

In conclusion, this study revealed that propranolol could improve OS in patients with unresectable/metastatic HCC. Propranolol could not only lower the risk of variceal bleeding and HCC development in patients with cirrhotic livers but also lower the mortality risk in those with advanced HCC. Future prospective studies based on ADRB2 expression in HCC patients are warranted to identify who would benefit most from propranolol treatment.

## Supporting information

S1 Table Multivariate stratified Cox regression analysis of mortality in patients with unresectable metastatic HCC. (DOC)

S2 Table Joint analysis of the effect of propranolol in combination with sorafenib, TACE, or radiotherapy on HCC mortality. (DOC)

S3 Table Multivariate Cox regression analysis of all-cause/ cancer-specific/ HCC mortality in patients with unresectable metastatic HCC. (DOC)

S4 Table Number of mortality. (DOC)
