# Supplementary material for: The effect of propranolol on the prognosis of hepatocellular carcinoma: A nationwide population-based study
Source: PLoS One. 2019 May 24;14(5):e0216828. doi: 10.1371/journal.pone.0216828 (PMC6534323; doi:10.1371/journal.pone.0216828)
Supplement: S1 Table — (DOC) [file pone.0216828.s001.doc]

S1 Table. Multivariate stratified Cox regression analysis of mortality in patients with unresectable metastatic HCC

|  | **Propranolol** | | | **Non-propranolol** | | |  | |
| --- | --- | --- | --- | --- | --- | --- | --- | --- |
| **Variable** | **Event** | **PY** | **Rate** | **Event** | **PY** | **Rate** | **Adjusted HR (95% CI)** | **P** |
| Total | 918 | 1812.77 | 50.64 | 2292 | 3472.18 | 66.01 | 0.78 (0.72-0.84) | <0.001* |
| **Gender** | | | | | | | | |
| Male | 691 | 1257.92 | 54.93 | 1667 | 2395.28 | 69.60 | 0.80 (0.74-0.86) | <0.001* |
| Female | 227 | 554.85 | 40.91 | 625 | 1076.90 | 58.04 | 0.71 (0.66-0.77) | <0.001* |
| **Age (years)** | | | | | | | | |
| ≥65 | 453 | 1021.29 | 44.36 | 1128 | 2010.31 | 56.11 | 0.80 (0.74-0.87) | <0.001* |
| 50-64 | 319 | 571.22 | 55.85 | 812 | 1079.73 | 75.20 | 0.75 (0.79-0.81) | <0.001* |
| 20-49 | 146 | 220.25 | 66.23 | 352 | 382.14 | 92.11 | 0.73 (0.67-0.79) | <0.001* |
| **HBV** | | | | | | | | |
| With | 267 | 611.91 | 43.63 | 644 | 1341.14 | 48.02 | 0.92 (0.85-0.99) | 0.045* |
| Without | 651 | 1200.85 | 54.21 | 1648 | 2131.04 | 77.33 | 0.71 (0.66-0.77) | <0.001* |
| **HCV** | | | | | | | | |
| With | 215 | 665.59 | 32.30 | 520 | 1357.97 | 38.29 | 0.85 (0.78-0.92) | 0.001* |
| Without | 703 | 1147.18 | 61.28 | 1772 | 2114.21 | 83.81 | 0.74 (0.69-0.81) | <0.001* |
| **Alcoholic liver disease** | | | | | | | | |
| With | 64 | 90.43 | 70.77 | 143 | 229.43 | 62.33 | 1.15 (0.98-1.50) | 0.07 |
| Without | 854 | 1722.34 | 49.58 | 2149 | 3242.75 | 66.27 | 0.76 (0.70-0.82) | <0.001* |
| **Liver cirrhosis** | | | | | | | | |
| With | 556 | 1066.61 | 52.13 | 1,499 | 2222.0 | 67.46 | 0.78 (0.72-0.85) | <0.001* |
| Without | 362 | 746.16 | 48.52 | 793 | 1250.18 | 63.43 | 0.78 (0.72-0.84) | <0.001* |
| **Renal failure** | | | | | | | | |
| With | 47 | 64.73 | 72.61 | 92 | 138.48 | 66.43 | 1.11 (0.92-1.21) | 0.18 |
| Without | 871 | 1748.04 | 49.83 | 2200 | 3333.70 | 65.99 | 0.77 (0.71-0.83) | <0.001* |
| **DM** | | | | | | | | |
| With | 192 | 344.05 | 55.81 | 392 | 605.67 | 64.72 | 0.87 (0.81-0.94) | 0.008* |
| Without | 726 | 1468.71 | 49.43 | 1900 | 2866.51 | 66.28 | 0.76 (0.70-0.82) | <0.001* |
| **HTN** | | | | | | | | |
| With | 174 | 326.19 | 53.34 | 296 | 519.92 | 56.93 | 0.95 (0.88-1.03) | 0.30 |
| Without | 744 | 1486.57 | 50.05 | 1996 | 2952.26 | 67.61 | 0.75 (0.69-0.81) | <0.001* |
| **CAD** | | | | | | | | |
| With | 22 | 64.73 | 33.99 | 53 | 138.48 | 38.27 | 0.90 (0.83-0.97) | 0.025* |
| Without | 896 | 1748.04 | 51.26 | 2239 | 3333.70 | 67.16 | 0.77 (0.72-0.84) | <0.001* |
| **Aspirin** | | | | | | | | |
| With | 177 | 798.80 | 22.16 | 398 | 1478.57 | 26.92 | 0.83 (0.77-0.90) | <0.001* |
| Without | 741 | 1013.97 | 73.08 | 1894 | 1993.61 | 95.00 | 0.78 (0.72-0.84) | <0.001* |
| **Statins** | | | | | | | | |
| With | 15 | 125.45 | 11.96 | 30 | 297.01 | 10.10 | 1.20 (0.99-1.38) | 0.07 |
| Without | 903 | 1687.32 | 53.52 | 2262 | 3175.17 | 71.24 | 0.76 (0.70-0.82) | <0.001* |
| **Metformin** | | | | | | | | |
| With | 135 | 327.09 | 41.27 | 270 | 564.49 | 47.83 | 0.87 (0.81-0.94) | 0.001* |
| Without | 783 | 1485.68 | 52.70 | 2022 | 2907.69 | 69.54 | 0.77 (0.71-0.83) | <0.001* |
| **Fibrates** | | | | | | | | |
| With | 170 | 286.46 | 59.35 | 361 | 497.78 | 72.52 | 0.83 (0.77-0.90) | <0.001* |
| Without | 748 | 1526.31 | 49.01 | 1931 | 2974.40 | 64.92 | 0.77 (0.71-0.83) | <0.001* |
| **TZDs** | | | | | | | | |
| With | 157 | 301.79 | 52.02 | 324 | 590.24 | 54.89 | 0.96 (0.89-1.04) | 0.13 |
| Without | 761 | 1510.98 | 50.36 | 1968 | 2881.94 | 68.29 | 0.75 (0.69-0.81) | <0.001* |
| **ACEIs** | | | | | | | | |
| With | 40 | 552.46 | 7.24 | 97 | 1097.80 | 8.834 | 0.83 (0.77-0.90) | <0.001* |
| Without | 878 | 1260.31 | 69.67 | 2195 | 2374.38 | 92.45 | 0.76 (0.71-0.82) | <0.001* |

*Significantly correlated with outcome, P-value < 0.05. HCC, hepatocellular carcinoma; PY, person-years; Rate, per 1000 person-years; HR, hazard ratio; CI, confidence interval; HBV, hepatitis B virus; HCV, hepatitis C virus; DM, diabetes mellitus; HTN, hypertension; CAD, coronary artery disease; TZDs, thiazolidinediones; ACEIs, angiotensin-converting enzyme inhibitors
