# Supplementary material for: The effect of propranolol on the prognosis of hepatocellular carcinoma: A nationwide population-based study
Source: PLoS One. 2019 May 24;14(5):e0216828. doi: 10.1371/journal.pone.0216828 (PMC6534323; doi:10.1371/journal.pone.0216828)
Supplement: S2 Table — (DOC) [file pone.0216828.s002.doc]

S2 Table. Joint analysis of the effect of propranolol in combination with sorafenib, TACE, or radiotherapy on HCC mortality

|  | **Adjusted HR (95% CI)** | **P** |
| --- | --- | --- |
| **Sorafenib** |  |  |
| **Sorafenib (-), Propranolol (-)** | Reference |  |
| **Sorafenib (-), Propranolol (+)** | 0.78 (0.58-0.99) | 0.022* |
| **Sorafenib (+), Propranolol (-)** | 0.65 (0.43-0.82) | 0.001* |
| **Sorafenib (+), Propranolol (+)** | 0.43 (0.21-0.61) | <0.001* |
| **TACE** |  |  |
| **TACE (-), Propranolol (-)** | Reference |  |
| **TACE (-), Propranolol (+)** | 0.79 (0.57-0.99) | 0.023* |
| **TACE (+), Propranolol (-)** | 0.80 (0.73-0.97) | 0.025* |
| **TACE (+), Propranolol (+)** | 0.52 (0.40-0.61) | <0.001* |
| **Radiotherapy** |  |  |
| **Radiotherapy (-), Propranolol (-)** | Reference |  |
| **Radiotherapy (-), Propranolol (+)** | 0.79 (0.59-0.99) | 0.021* |
| **Radiotherapy (+), Propranolol (-)** | 0.98 (0.72-1.29) | 0.315 |
| **Radiotherapy (+), Propranolol (+)** | 0.70 (0.52-0.97) | 0.013* |

*Significantly correlated with outcome, P-value < 0.05. TACE, transarterial chemoembolization; HCC, hepatocellular carcinoma; HR, hazard ratio; CI: confidence interval
