# Supplementary material for: The effect of propranolol on the prognosis of hepatocellular carcinoma: A nationwide population-based study
Source: PLoS One. 2019 May 24;14(5):e0216828. doi: 10.1371/journal.pone.0216828 (PMC6534323; doi:10.1371/journal.pone.0216828)
Supplement: S3 Table — (DOC) [file pone.0216828.s003.doc]

S3 Table. Multivariate Cox regression analysis of all-cause / cancer-specific / HCC mortality in patients with unresectable metastatic HCC

|  | **Propranolol** *vs.* **Non-propranolol** *(Reference)* | |
| --- | --- | --- |
| **Propensity score matching** | **1 : 1** | **1 : 2** |
| **Events** | **Adjusted HR (95% CI)** | **Adjusted HR (95% CI)** |
| All-cause mortality | 0.80 (0.73-0.85)* | 0.78 (0.72-0.84)* |
| Cancer-specific mortality | 0.73 (0.69-0.82)* | 0.72 (0.68-0.80)* |
| HCC mortality | 0.74 (0.70-0.82)* | 0.72 (0.69-0.81)* |

*Significantly correlated with outcome, P-value < 0.05. HCC, hepatocellular carcinoma; HR, hazard ratio; CI, confidence interval
