# Supplementary material for: The effect of propranolol on the prognosis of hepatocellular carcinoma: A nationwide population-based study
Source: PLoS One. 2019 May 24;14(5):e0216828. doi: 10.1371/journal.pone.0216828 (PMC6534323; doi:10.1371/journal.pone.0216828)
Supplement: S4 Table — (DOC) [file pone.0216828.s004.doc]

S4 Table. Number of mortality

|  | **Propranolol** | **Non-propranolol** |
| --- | --- | --- |
| All-cause mortality | 918 | 2,292 |
| Cancer-specific mortality | 824 | 2,065 |
| HCC mortality | 799 | 1,996 |
